# Triplin: Mechanistic Basis for Voltage Gating

**DOI:** 10.3390/ijms241411473

**Published:** 2023-07-14

**Authors:** Marco Colombini, Patrick Liu, Chase Dee

**Affiliations:** Department of Biology, University of Maryland, College Park, MD 20842, USA

**Keywords:** voltage dependence, voltage sensor, porin, prokaryote, kinetics, polyarginine, cooperativity, pore

## Abstract

The outer membrane of Gram-negative bacteria contains a variety of pore-forming structures collectively referred to as porins. Some of these are voltage dependent, but weakly so, closing at high voltages. Triplin, a novel bacterial pore-former, is a three-pore structure, highly voltage dependent, with a complex gating process. The three pores close sequentially: pore 1 at positive potentials, 2 at negative and 3 at positive. A positive domain containing 14 positive charges (the voltage sensor) translocates through the membrane during the closing process, and the translocation is proposed to take place by the domain entering the pore and thus blocking it, resulting in the closed conformation. This mechanism of pore closure is supported by kinetic measurements that show that in the closing process the voltage sensor travels through most of the transmembrane voltage before reaching the energy barrier. Voltage-dependent blockage of the pores by polyarginine, but not by a 500-fold higher concentrations of polylysine, is consistent with the model of pore closure, with the sensor consisting mainly of arginine residues, and with the presence, in each pore, of a complementary surface that serves as a binding site for the sensor.

## 1. Introduction

**Figure 1:**
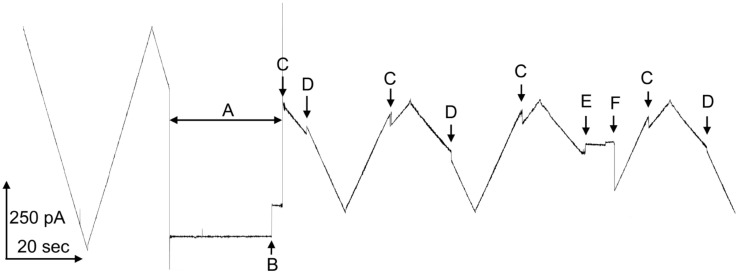
Voltage gating in a single Triplin. On the left side, a triangular voltage wave (+75/−77mV; 30 mHz) results in no pore closure. In region “A”, a +69 mV potential was applied to the *cis* compartment. At point “B”, pore 1 closed. The resumption of the triangular wave caused gating of pores 2 and 3. “C” indicates the location of pore 2 closure and “D” the locations of pore 2 reopening. “E” is the point at which pore 3 closed and at “F” pores 2 and 3 opened simultaneously.

Triplin is a channel-forming structure found in *Escherichia coli*. It forms a set of three pores whose effective size and conductance resemble OmpC, whereas its weak ion selectivity resembles OmpF [1]. Triplin differs dramatically for all porins described to date (e.g., [2,3,4,5]) in that it displays steep voltage dependence [6] comparable to that of the voltage-gated channels responsible for the electrical excitability of the mammalian nervous system. A unique property of Triplin that distinguishes it from all membrane channels and pores described to date is the remarkable interpore cooperative behavior in that pore 1 must close first, its closure then allows pore 2 to close and, in turn, pore 2 closure allows pore 3 to close [6]. This is illustrated in Figure 1. At left, a single Triplin shows no voltage-dependent gating. The application of a high positive potential during time interval “A”, closes pore 1 (at point “B”) and it remains closed. That closure allows pore 2 to close at negative voltages (at point “C”). The reopening of pore 2 (at point “D”) prevents the closing of pore 3 (at point “E”). A delayed reopening of pore 2 allows pore 3 to close at positive voltages.

Insights into the molecular basis for this complex were obtained by probing the nature and dynamics of the voltage sensors of Triplin [1]. This led the authors to propose the following model.

**Figure 2:**
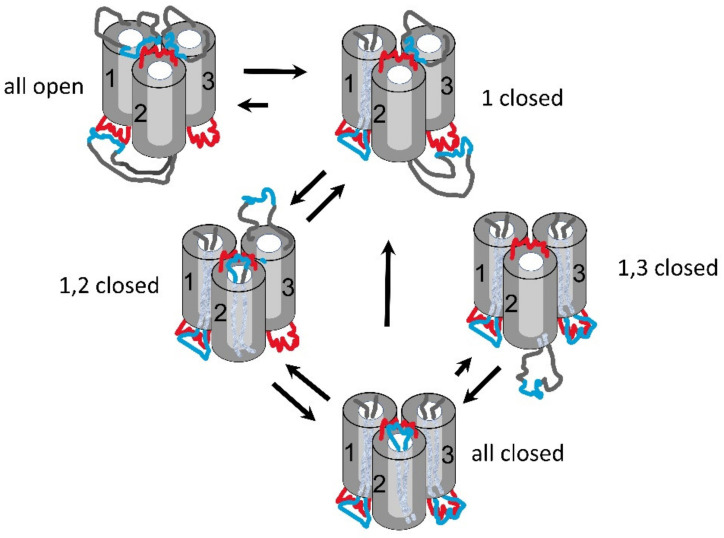
Model of the gating of Triplin. The top of the structure is the *cis* side of the membrane, the side from which Triplin is inserted. The bottom of the structure is the *trans* side and that is the side maintained at virtual ground by the amplifier. The numbers refer to pores 1, 2 and 3. All indicated voltages refer to the *cis* side. For simplicity, the closed state of the pore is illustrated as a result of blockage by a single loop of the beta barrel, but, of course, multiple loops may be involved. Blue regions are positively charged whereas red are negatively charged. From Figure 14 in ref [1].

Triplin is proposed to form three beta barrel pores, similar to those of other three-pore porins, but one pore-forming structure is oriented opposite to the others [1]. In the model (Figure 2), each pore-forming subunit has a positively charged voltage sensor (blue region) located in external loops on one end of the pore. Pore closure is proposed to result from the entry of the sensor into the pore, thus blocking ion flow. When the sensor is in the pore, it is proposed to interact with a negative domain located at the other end of the pore, with the negative domain also being responsible for the pore’s weak cation selectivity and rectification [1]. No evidence was presented to support this closing mechanism, and this paper addresses that issue.

## 2. Results

### 2.1. Kinetic Measurements

#### 2.1.1. Kinetic Measurements Can Distinguish between Viable Gating Mechanisms

The molecular mechanism for the voltage gating of the pore structures formed by Triplin assumes that these are formed by beta barrels. The similarity of the conductance and selectivity of the Triplin pores to those of porins like OmpF and OmpC whose structure is well established, makes the assumption of a beta barrel very likely to be correct. The steep voltage dependence of the Triplin pores, requiring the translocation of 14 charges across the membrane [6], only leaves 2 mechanisms: translocation of charges through the lipid bilayer or translocation through the hole formed by the pore. Other models for the gating of porins, such as the movement of a loop located withing the pore itself, are not tenable. Whereas the movement of such a loop could easily obstruct the flow of ions, it would not be able to translocate sufficient charge to account for the steep voltage dependence. As for the two viable models, they make different predictions on the kinetics of the gating process. Translocation of charges through the lipid bilayer should result in the same voltage dependence of the rate constant for pore opening and closure because the energy barrier for both processes is the energy required to insert a highly charged domain into the hydrophobic portion of the membrane, and thus, the barrier height should be the same. If the sensor enters the pore and binds to the proposed negative domain at the other end of the pore, then it may travel through a large portion of the electric field before reaching the energy barrier. Thus, kinetic studies could distinguish between the two processes.

#### 2.1.2. Measurement of the Kinetics of the Gating of Pore 2

The only pore-forming subunit amenable to kinetic measurements was pore 2. The kinetics of pore 1 are too slow. Pore 3 gating depends on pore 2 being closed, and performing kinetic measurements in the positive voltage region leads to pore 2 opening. Another limitation is the necessity to perform these measurements on single Triplins. In a multi-Triplin experiment, one cannot be certain that the gating of the pores of other Triplins would not influence the data collected. The data on the reopening of pore 2 was influenced by the closing of pore 3 at the higher positive voltages (Section 2.1.3) but that can be corrected.

**Figure 3:**
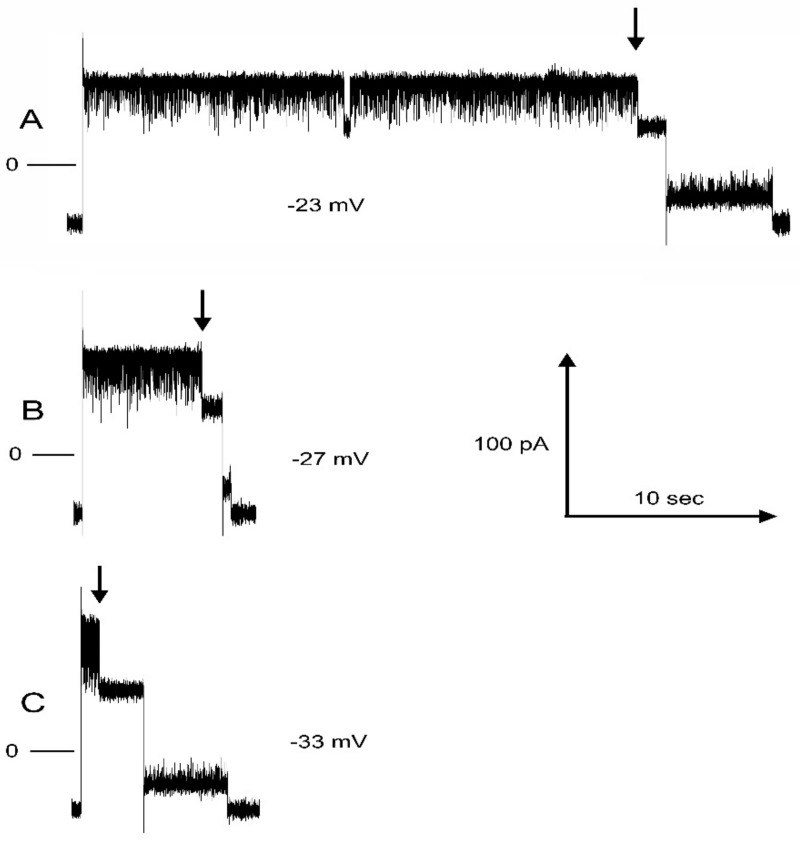
Pore 2 closing time at three different voltages, labeled (**A**–**C**). In all records, the initial voltage was 10 mV (short segment on left). The voltage was then switched to the indicated value, and at the point indicated by the arrow, pore 2 closed. The voltage was then switched to 10 mV, and shortly thereafter, the pore reopened. Zero current is indicated by the short line on the left side. The inset shows a plot of the average closure time (the time constant) as a function of voltage for that experiment.

Figure 3 shows examples of single records of the time for pore 2 closure at three negative voltage values. Note that the pore is flickering to an unstable closed state, and the closing time measured was the time to reach a stable closed state indicated by the arrow and the current remaining stable at the level at which only pore 3 is conducting. The times for closure varied stochastically as expected for single-molecule experiments. The mean time of typically 15 recordings was used to obtain a reliable value for the closing time constant, τ. The inset shows how τ varied with the applied voltage for one complete experiment.

**Figure 4:**
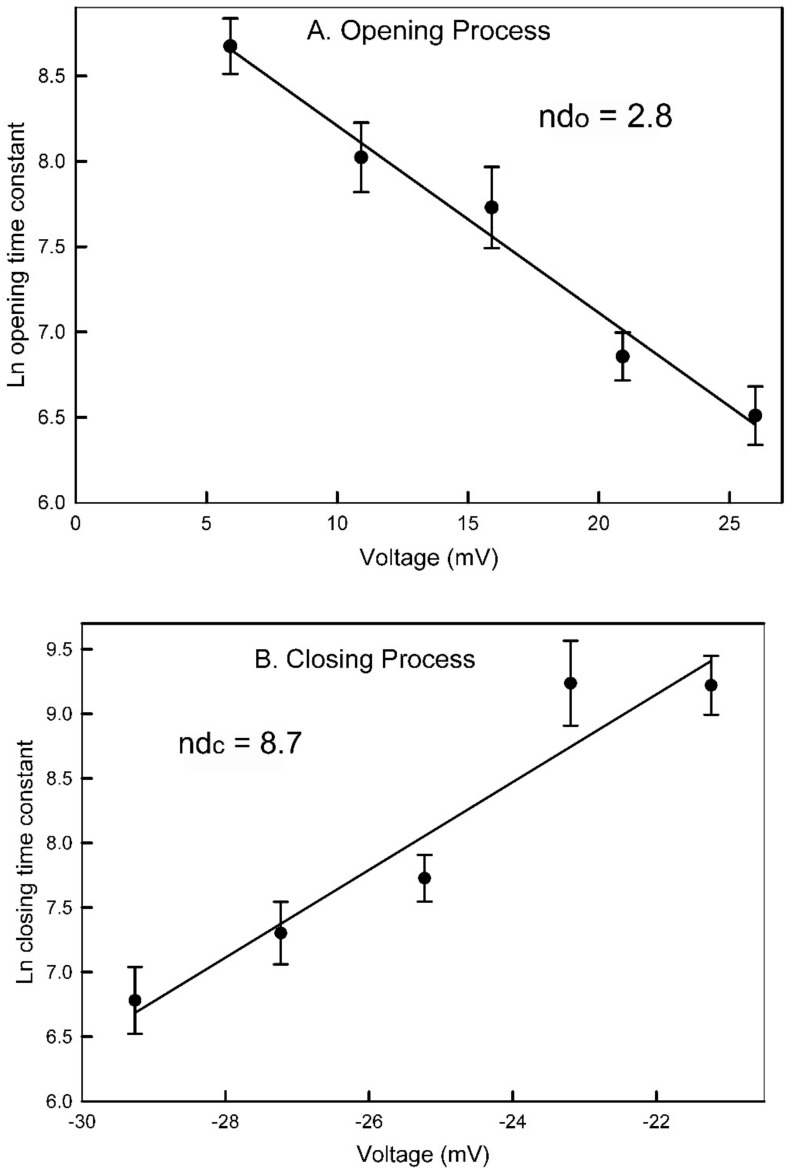
Voltage dependence of the opening and closing time constants for pore 2. The error bars are standard errors of the mean of 20 measurements.

As described by the theory in the Section 5, the log transform of τ was then used to obtain an estimate of the fraction of the electric field, “*d*”, traversed by the sensor when going either from the closed state, *d_o_*, or from the open state, *d_c_*, to the peak of the energy barrier (Figure 4). The energy change that determines τ is a product of the charge moved and the voltage change that takes place when the sensor reaches the peak of the energy barrier. The slope of the fit lines is equal to ndF/RT, the energy difference divided by thermal energy. When the slope is multiplied by RT/F, it yields the product of the number of gating charges times the fraction of the field traversed, *nd_c_* and *nd_o_*, depending on whether the data collected was for the closure or opening process, respectively. The summation of *nd_c_* and *nd_o_* yields the effective number of gating charges, *n*, which for this experiment was 11.5. The average of six independent experiments resulted in *nd_c_* = 8.7 ± 0.7 and *nd_o_* = 2.5 ± 1.2 (mean ± SD). That yields a value for *n* of 11.2. Thus, *d_c_* = 0.78 and *d_o_* = 0.22.

The blockage of the ion flow through the pore will change the shape of the transmembrane electric field, and so hard conclusions can only be made if the whole process was understood in detail. Regardless, the sensor is moving through a major portion of the electric field before reaching the peak energy barrier, and this is consistent with the sensor moving into the pore eventually obstructing the pore. The charged region is leading the way and thus translocating though a large portion of the electric field.

#### 2.1.3. Kinetics Insights into Interpore Control

**Figure 5:**
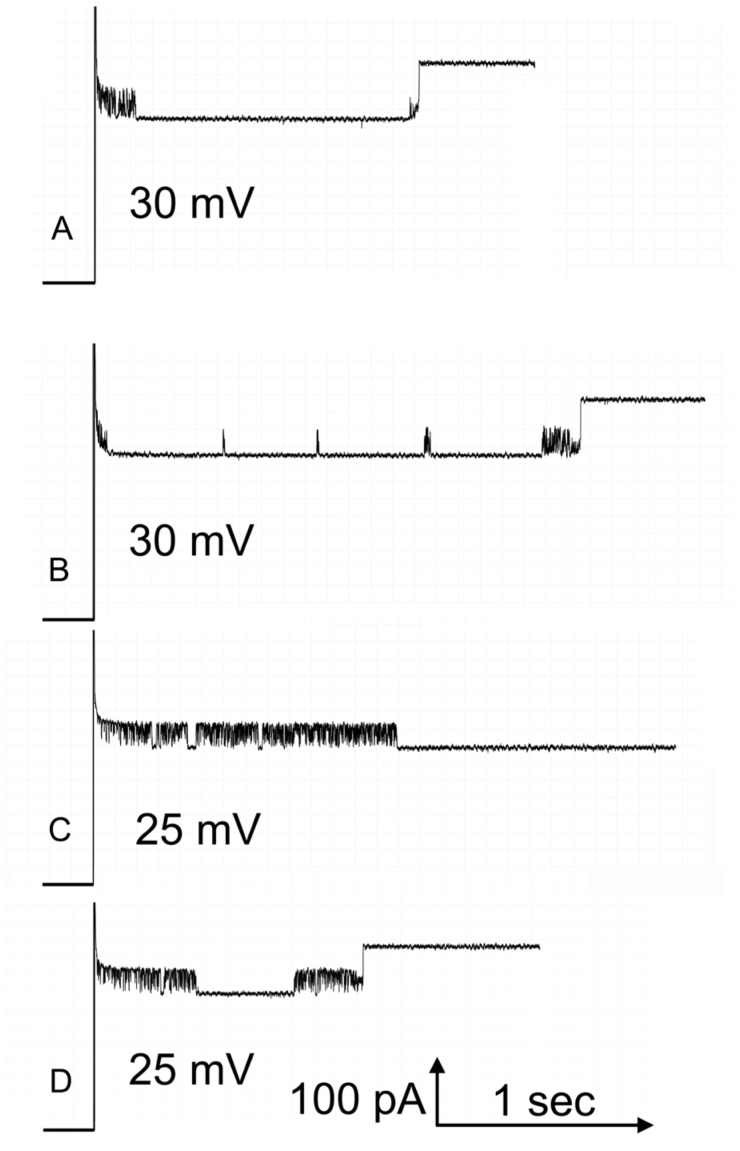
Pore 3 closure interferes with the opening of pore 2. Four sample records are illustrated, two taken at 25 mV (**C**,**D**) and two at 30 mV (**A**,**B**). Pore 2 was closed at −40 mV, and then the positive voltage indicated was applied. The downward events are pore 3 closures: some are transient and other are long-lived. In the case of record (**C**), the reopening of pore 3 after its closure in the middle of the record, took place at a time beyond the end of the record shown. Hence, pore 2 opening is not visible in the record shown.

**Figure 6:**
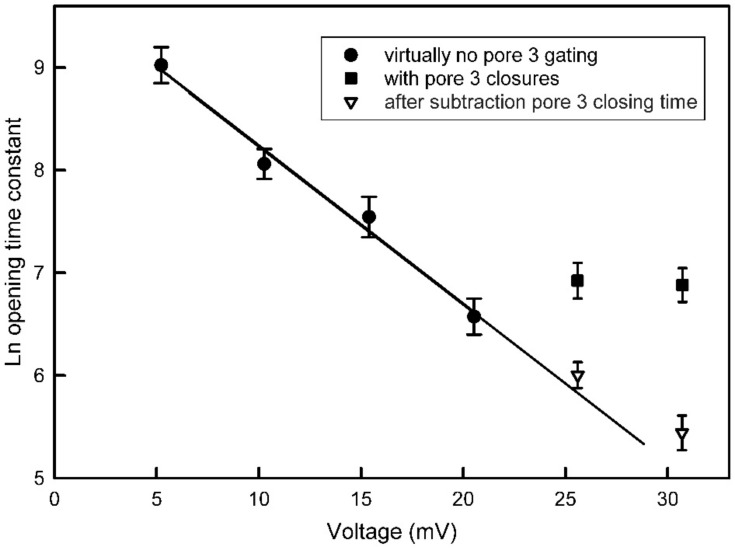
Pore 3 closure blocks pore 2 opening. Pore 2 opening at various applied voltages was measured either by ignoring pore 3 closure (circle and square symbols) or by subtracting the time during which pore 3 was closed (triangles). Error bars are SEM of 18 to 22 measurements.

The opening process for pore 2 takes place at a manageable rate at positive voltages (see Figure 4). However, the closing process for pore 3 also takes place at positive voltages. Figure 5 shows examples of the opening of pore 2 at 25 mV and 30 mV. Pore 2 was closed at −40 mV and then the voltage switched to the indicated value. What is taking place is most clearly seen in traces recorded at 25 mV where the closure of pore 3 is less frequent. Focusing on trace “D”, there are both short-lived and one long-lived drops in current due to pore 3 closure. Finally, pore 2 opens resulting in a current increase that is double the lowest current value in the record and shows reopening of both pores 2 and 3. Note that there is no closure of pore 3 after pore 2 opening. In the traces recorded at 30 mV (traces “A” and “B”), pore 3 is closed most of the time, inhibiting the opening of pore 2. At higher positive voltage, the rate of opening of pore 2 should be faster, and it is if the time pore 3 is closed is subtracted from the total time. If the pores were functioning independently, then the probability of pore 2 opening and that of pore 3 closing should take place regardless of the state of the other pore. Figure 6 shows, quantitatively, how the rate of pore 2 opening is greatly slowed down at the higher positive voltages if one does not correct for the time that pore 3 is closed. By subtracting the time when pore 3 is closed, the data follows the expected linear relationship of the log plot. Thus, the kinetic measurements also demonstrate the interdependence of the gating of Triplin’s pores.

### 2.2. Pore Blockage by Polyarginine

Evidence indicates that the sensors responsible for the closure of the Triplin pores must contain a minimum of 14 positive charges and most of those are likely to be arginines [1]. If, as proposed, a sensor were to physically block a pore by inserting into the pore, it is reasonable to conclude that evolutionary adaptation of the process would result in a binding surface that complements the physical nature of the sensor. Thus, polyarginine might be sufficiently similar to the sensor so as to mimic its ability of blocking the pores. Experiments using polyarginine, with an average molecular mass of 10,000, show that blockage can begin to be measured at a concentration in the solution next to the membrane of 40 nM. This signifies strong binding. The same experiments using polylysine with an average molecular mass of 15,000 resulted in no blockage even with a final concentration of 23 µM. 800 µM spermine (a polyamine containing four amines) also had no blocking effect. Thus, it appears that there is a surface complementary to the sensor, and it is specific for polyarginine.

**Figure 7:**
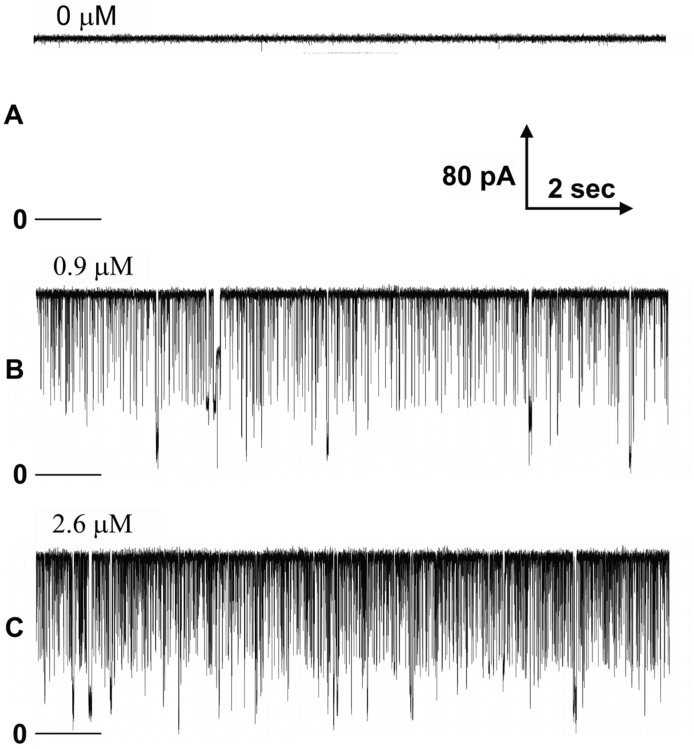
Transient blocking events by the addition of the indicated amount of polyarginine to the cis side of a membrane containing a single Triplin with all pores open. The three records shown (**A**–**C**) are typical samples of the recorded blockage events. The zero current level is indicated just below the record. All records were collected in the presence of a 40 mV applied potential.

**Figure 8:**
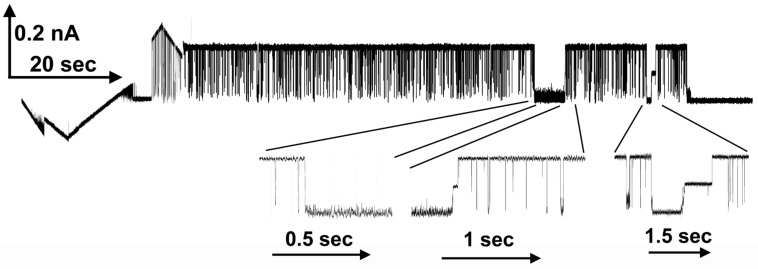
Long-lived pore blockage in the presence of 0.4 µg/ml polyarginine. A single Triplin was still gating as demonstrated by the triangular voltage wave (+72/−71 mV) on the far left side. Pore 2 closure was followed by pore 3 closure and then simultaneous pore 2 and 3 opening at high positive voltages allowing polyarginine to block transiently. This was followed by applying a constant voltage (+50 mV) resulting in both transient and long-lived blockages. The expanded regions show that often both pores were blocked simultaneously but at times unblocking took place in two separate events. These regions were only expanded in the time axis.

Figure 7 shows the transient blockages observed when +40 mV was applied to the cis compartment of a membrane containing a single Triplin. Records were taken prior to and after sequential additions of polyarginine. Transient blockages were observed only at positive potentials as expected since a positive potential would drive polyarginine into the pores. In this record, all three pores were open and were blocked, sometimes individually, but often two at a time or all at once. This is seen more clearly in Figure 8. In the illustrated portion of this experiment, pore 1 was closed and pores 2 and 3 gated normally when a triangular voltage wave was applied (far left). The low dose of polyarginine used in this experiment did not induce pore 1 opening. The application of a constant +50 mV resulted in frequent fast blockage but also two periods of prolonged blockage. The simultaneous blockage of both pores was often followed by separate pore unblockage. On the right side of the figure, the blockage was persistent, and a negative potential was necessary to unblock the pores and repeat the blocking experiment. The simultaneous blockage of two and three pores is easily explained by considering that the long polyarginine chain (average of about 65 residues) can straddle two and three pores at the same time.

**Figure 9:**
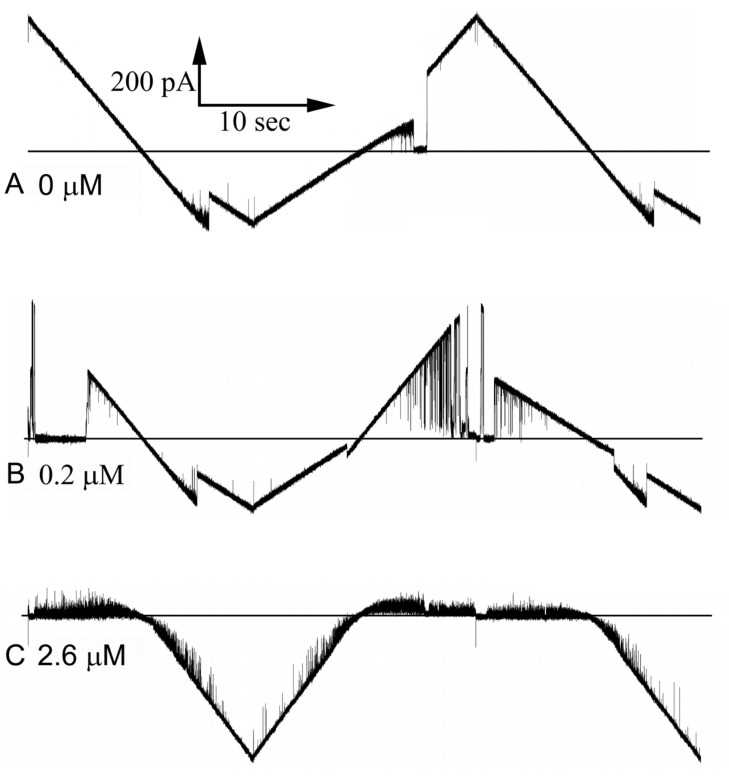
Voltage-dependent block of Triplin by polyarginine. A triangular voltage wave (30 mHz; +72 to −71 mV) was applied to a membrane containing a single Triplin activated by closing pore 1. The horizontal lines are the zero current levels. Polyarginine was added sequentially, and sample records are illustrated. Record (**A**) was taken before polyarginine addition, followed later by (**B**) and much later by (**C**). The amount of polyarginine present in the cis compartment during each recording is indicated.

Figure 9 shows current recordings as triangular voltage waves were applied. Note that for high doses of polyarginine (trace “C”) pore 1 opened thus increasing the conductance at negative voltages. Blocking occurred only at positive voltages on the side of the membrane to which polyarginine was added. Negative voltages unblock the pore including long-lived blocking events that are more common at high concentrations of polyarginine.

**Figure 10:**
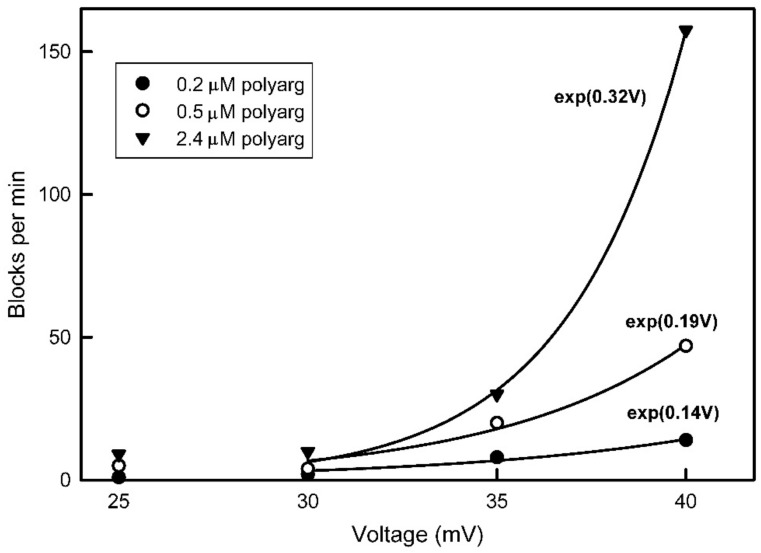
Voltage dependence of the formation of long-lived pore blocks by polyaginine at the indicated concentration. The power dependence of the exponential fit to the data beginning at 30 mV is indicated next to each curve.

Transient blockages increase weakly with voltage whereas long-lived blockages are far more voltage dependent. The frequency of such blockage increases exponentially with applied voltage (Figure 10). Increasing voltages also increases the occurrence of blockages that require a negative voltage to unblock.

## 3. Discussion

Triplin is a three-pore complex, each pore possessing a positively charged voltage sensor consisting of 14 net charges [1,6]. Each sensor domain translocates across the membrane in a steeply voltage-dependent manner, and this translocation is coupled with the closure of each pore [1]. The proposed molecular mechanism by which sensor translocation results in pore closure is for the sensor to enter the pore and obstruct the flow of ions [1]. With an estimated pore diameter of 0.9 nm [1], there is enough space for surface loops to enter the pore and obstruct it. Indeed, it is not uncommon for porin to normally have a surface loop inserted into the pore, reducing its effective diameter (e.g., loop 3 in OmpF [5]). Other mechanisms are possible [7], but the experimental evidence presented here supports the proposed mechanism.

Kinetic measurements can not only provide insight into the molecular mechanism by which voltage-gating takes place but, more importantly, can provide compelling evidence against the proposed mechanism. The kinetics of a well-studied beta barrel channel former, VDAC, provide useful contrasting kinetic properties to Triplin. For Triplin, the rates of pore closure for pores 2 and 3 are fast whereas the rates of reopening are slow [6]. For VDAC, pore closure is slow (τ ≈ 400 s) and voltage dependent whereas reopening is fast (τ ≈ 2.5 ms) and voltage independent [8]. The voltage-dependent effective closure of VDAC (actually a reduction in pore size and selectivity inversion that results in no flux of ATP) is achieved by the translocation of portions of the beta barrel being moved to the membrane surface [8]. The slow closure rate of VDAC can be readily understood by the need to break at least two sets of hydrogen bonds that anchor the mobile domain to the rest of the transmembrane beta barrel before they can translocate to the membrane surface. Reopening involves moving this mobile region from the membrane surface and reinserting it into the beta barrel. Half as many hydrogen bonds in the beta barrel would need to be broken for the reinsertion of the mobile domain to take place.

**Figure 11:**
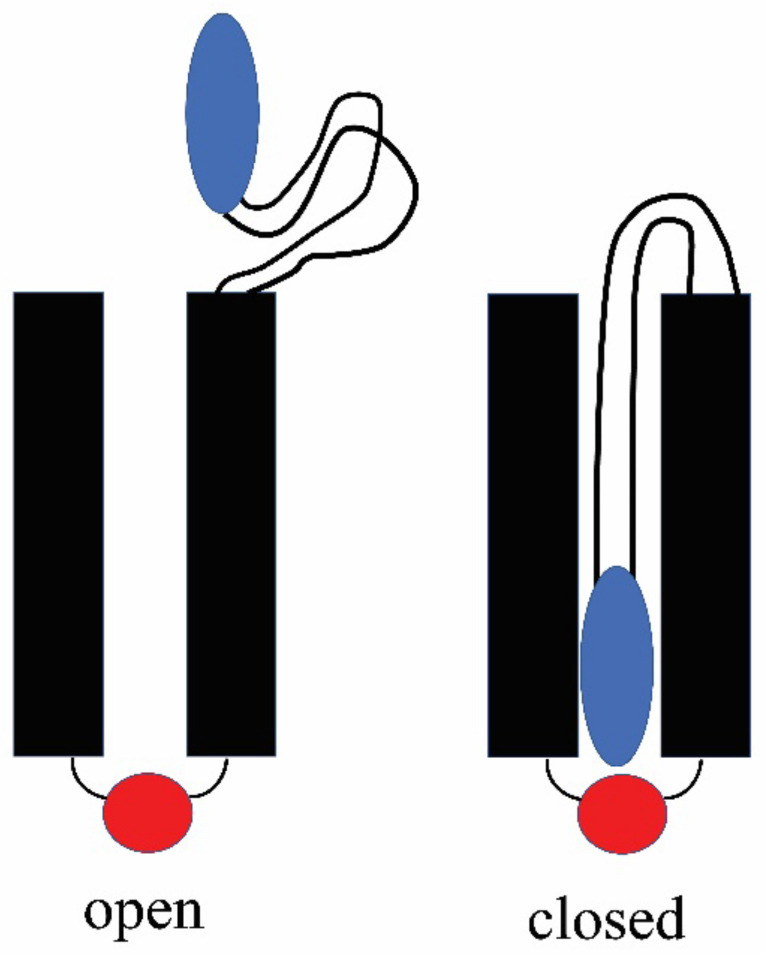
Model of the gating process used by each pore. Left is the open state with the sensor (blue) out of the pore. The red negative domain is close to the other end of the pore and proposed to be responsible for the selectivity of the pore and the rectification. Right shows the obstructed pore with the sensor (blue) interacting with a negative domain (red).

For Triplin, the proposed mechanism would reverse the energetics. Closure of the pores in Triplin is proposed to result from the entry of a surface loop-like domain into the pore led by the positively charged region (blue; Figure 11). Once in the pore, this mobile sensor domain is proposed to bind to a negatively charged domain (red) on the other end of the pore. The breaking of that electrostatic interaction then would be responsible for the slow reopening kinetics.

The measured voltage dependence of pore-closing and opening time constants provides independent support for the pore-blocking closure mechanism. The pore-closing process is four times more voltage dependent than the opening process indicating that the energy barrier for the process is deep within the pore. Therefore, the closing process would require the sensor to move through most of the electric field before reaching the peak of the energy barrier, and the reverse would be the case for the reopening process. Focusing on the gating model, what aspect of the structure might correspond to the energy barrier? All surfaces, especially charged surfaces, have a layer of bound water of hydration. This is known to be a critical factor in determining the binding energy between two surfaces and thus critical to determining the binding selectivity as demonstrated by George Eisenman [9]. Thus, the location of the energy barrier could very well be at a point where the sensor approaches the complementary binding domain. If the proposed negatively charged region at the opposite end of the pore was part of that complementary surface, it would explain (correlate with) the results of the kinetic studies.

The existence of a binding region complementary to the sensor domain is strongly supported by the specificity of the polyarginine blocking. The differential ability to block the pore by polyarginine as opposed to polylysine is greater than 500-fold since no detectable blocking by polylysine was observed. This specificity of the blocking of polyarginine over polylysine is not related to any propensity for secondary structure formation as both have an extended conformation under the experimental conditions used [10,11]. Thus, the very strong preference for polyarginine indicates a specific interacting surface in Triplin that goes beyond just an electrostatic interaction.

Is the blockage of ion flow through the Triplin pores really mimicking the blockage produced by the sensor domain? Whereas the long-lived blockages must be physical blockages, the transient ones might be the result of polyarginine translocating through the pore as it moves from one side of the membrane to the other. However, calculations indicate that an extended polyarginine chain driven through the pore with an electric field should travel at a rate that is 10^4^ times faster than that observed for the typical duration of the fast current blocking (flickering) events. Thus, it is more likely that the flickering is a result of transient blocking of the pore. This conclusion is supported by the observation that frequently all the conductance is blocked simultaneously, and thus, all open pores of a Triplin are blocked simultaneously. That would be a very unlikely event if the conductance drops were due to polyarginine translocating through the pores.

An observation that would seem to indicate that the polyarginine is translocating through the pores rather than blocking is the finding that the flickering rate increases with an increase in the aqueous concentration of polyarginine. However, the interaction between polyarginine and Triplin may be quite labile, undergoing a dynamic equilibrium with dissolved polymer. Thus, increasing the concentration of polyarginine would increase the blocking frequency.

The properties of the polyarginine block are consistent with the model of the gating mechanism. The long, highly charged polypeptide chain should easily flow through a simple cylindrical pore, especially when driven by an electric field. Published work indicates that polyarginine should have a primarily extended conformation under the conditions of neutral pH used in these experiments [10]. Thus, the observation of blockage indicates some stabilizing interaction within the pore and/or interference to flow by the proposed negatively charged domain at one end of each pore.

## 4. Materials and Methods

### 4.1. Sources of Materials Used

All chemicals used were reagent grade. The phospholipids were obtained from Avanti Polar Lipids (Alabaster, AL, USA). Cholesterol, polylysine, polyarginine, and spermine were purchased from Sigma (St. Louis, MO, USA).

### 4.2. Electrophysiological Recordings

All experiments were performed on Triplin reconstituted into planar phospholipid membranes made from monolayers as described previously [12,13]. In brief, the membrane was formed across a 0.1 mm hole in a thin polyvinylidene chloride partition separating two aqueous compartments containing 5 mL of 1.0 M KCl, 1 mM MgCl_2_, buffered with 10 mM HEPES, pH 7.8. The monolayers were formed from a solution of 0.5% (*w*/*v*) diphytanoylphosphatidylcholine, 0.5% (*w*/*v*) polar extract of soybean phospholipids, and 0.05% (*w*/*v*) cholesterol in hexane. The hexane was allowed to evaporate prior to membrane formation, and thus, no solvents were present in the membrane. This membrane is identical to a natural cell membrane but lacks proteins or carbohydrates. Samples containing Triplin were generated as previously described [6], i.e., flash-frozen in 0.1 mL aliquots and stored at −80 °C. After thawing, β-octyl-glucoside was added to a final concentration of 1% (*w*/*v*) and kept on ice during the experiment. Typically, 10 µL of the sample was dispersed into one aqueous compartment (designated “*cis*”), and, with time, Triplin would insert into the membrane. The membrane voltage was clamped, and the current recorded using Clampex 10.3 software. Calomel electrodes were used to interface the solution with the electronics. Voltages was applied to the *cis* side, the *trans* held at virtual ground. The signal was low-pass filtered at 500 Hz. The sample containing Triplin was always added to the *cis* compartment. All measurements were performed at room temperature (23 to 24 °C)

### 4.3. Kinetic Measurements

Kinetic measurements were only made on pore 2 because the conditions necessary for making those measurements for pore 3 often resulted in the spontaneous opening of pore 2 and thus interfering with the collection of sufficient data to achieve valid measurements. Recordings were made on membranes containing a single Triplin so that the meaning of any conductance change was clearly defined. For the closing kinetics, pore 2 was held in the open state at 10 mV and then switched to a negative voltage until pore 2 closed, and thus, the time required for closure was recorded. This process was repeated 15 to 20 times, depending on the experiment, for negative voltages ranging from −20 to −30 mV. For the opening process, pore 2 was closed by applying −40 mV, and then, the voltage was switched to a positive value until the pore reopened. Again, the process was repeated 15 to 20 times, depending on the experiment, for positive voltages ranging from 5 to 40 mV. At the higher voltages, pore 3 would frequently close and reopen during the waiting period for pore 2 to reopen.

#### Theoretical Basis for Estimating the Fraction of the Electric Field Traversed Prior to Reaching the Peak of the Energy Barrier

Using Eyring Rate Theory, one can obtain an estimate of the fraction of the transmembrane electric field that the sensor needed to traverse prior to reaching the peak of the energy barrier.

pore closed ⇌ pore open

where the forward reaction rate constant is *k_o_* and the reverse is *k_c_*

$$\mathrm{mean}\;\mathrm{time}\;\mathrm{to}\;\mathrm{open}\;\mathrm{pore}=\tau_{o}=\frac{1}{k_{o}}$$

$$\mathrm{mean}\;\mathrm{time}\;\mathrm{to}\;\mathrm{close}\;\mathrm{pore}=\tau_{c}=\frac{1}{k_{c}}$$

From Eyring rate theory:

$$k_{c}=\frac{k_{B}T}{h}e^{\frac{-nd_{c}F(V-V_{0)}}{RT}}$$

where *h* is Plank’s constant, *k_B_* is Boltzmann’s constant, *n* is the number of charges on the voltage sensor, *d_o_* is the fraction of the electric field that the sensor must traverse to move from the closed state to the peak of the energy barrier whereas *d_c_* is from the open state to the barrier peak. *V* is the transmembrane voltage, and *V*_o_ is the voltage at which half the pores are open and half are closed. *R*, *T*, and *F* have their usual meanings.

After log transforming and simplifying:

$$\ln\left(\tau_{o}\right)=\frac{nd_{o}F}{RT}V+K_{o}$$

and

$$\ln\left(\tau_{c}\right)=\frac{nd_{c}F}{RT}V+K_{c}$$

## 5. Conclusions

The gating process used by each of the three pores formed by Triplin requires the movement of a highly positively charged domain from one side of the membrane to the other. Kinetic studies show that the sensor moves through most of the transmembrane electric field prior to reaching the peak of the interaction energy between the sensor and the pore structure. This agrees with the model that the sensor enters the pore and travels to the opposite side interacting with a complementary domain on the other side of the membrane and thus blocking the flow of ions and producing the closed state. In a recent publication [1], the authors proposed the existence of a negatively charged domain at one end of the pore just outside it as depicted in Figure 11. PNP calculations showed that such a structure would explain both the weak pore selectivity for cations and the observed rectification of the current flow through pores 1 and 3. This domain is very likely to be at least part of the complementary surface for the sensor. Being outside the pore, it would explain the reported [1] sensor cleavage by trypsin when the pore is closed. Polyarginine mimics the pore-blocking mechanism used by the sensor including voltage dependence of the polyarginine block. The inability of polylysine to block the pores supports the conclusion that the complementary surface binds arginines specifically, as opposed to a simple electrostatic interaction. This correlates with previous findings that the charges on the sensor are mainly arginines [1]. There is no alternative mechanism that is consistent with all the experimental observations.

## Data Availability

The data will be provided upon request of the corresponding author.
